# Prevalence of *Plasmodium* parasitaemia in blood donors and a survey of the knowledge, attitude and practices of transfusion malaria among health workers in a hospital in Kumasi, Ghana

**DOI:** 10.1371/journal.pone.0206303

**Published:** 2018-11-05

**Authors:** Kwame Agyemang Adusei, Alex Owusu-Ofori

**Affiliations:** 1 Medical Laboratory Technology, Kwame Nkrumah University of Science and Technology, Kumasi, Ghana; 2 Department of Clinical Microbiology, Kwame Nkrumah University of Science and Technology, Kumasi, Ghana; Université Pierre et Marie Curie, FRANCE

## Abstract

Malaria is one of the transfusion transmissible infections in malaria endemic countries such as Ghana. Healthy blood donors may harbour *Plasmodium* parasites without showing signs of malaria. Blood from such donors constitutes a risk to transfusion recipients and the recipients of this blood may go on to develop transfusion transmitted malaria (TTM). In many malaria endemic countries, blood donors are not screened for *Plasmodium* parasites. We investigated the prevalence of *Plasmodium* in blood donors in a hospital in Ghana as well as evaluate health workers knowledge, attitude and practices towards TTM. The study was carried out at the Kwadaso Seventh Day Adventist Hospital in Kumasi, Ghana from September 2016 to May 2017. Blood samples from 100 blood donors and 100 non-donors were examined for *Plasmodium* using microscopy and a rapid diagnostic test (RDT). In addition the blood groups of participants were determined. To obtain information concerning knowledge, attitude and practices of transfusion transmitted malaria, questionnaires were administered to 100 health workers including doctors, nurses and laboratory technicians. The prevalence rate of *Plasmodium* parasitaemia in blood donors by RDT and microscopy was 8% and 3% respectively, compared to non-donors who had a prevalence of 5% and 2% by RDT and microscopy respectively. Out of 100 health workers surveyed, 26% (26/100) had never heard of transfusion transmitted malaria. In an emergency situation, 41% health workers were willing to transfuse malaria positive blood but only 2%, 4% and 8% were willing to transfuse blood that was positive for HIV, Hepatitis B and Syphilis respectively. Regular training workshops may help improve the knowledge of health workers as a quarter of workers had not heard about transfusion transmitted malaria and 6.8% did not know that malaria was transmissible by transfusion.

## Introduction

Sub-Saharan Africa has a substantial burden of malaria disease with an estimated 90% of the world malaria-related deaths occurring in this territory. Twenty-five million pregnant women are currently at risk for malaria and according to the World Health Organization, malaria is responsible for over 200,000 neonatal and 10,000 maternal deaths per year[1]. In 2015, 429,000 deaths from malaria were estimated to occur globally, out of which 303,000 malaria deaths were estimated to occur in children aged under 5 years[2].

In the first half of the year 2016, Ghana recorded 4,940,270 suspected cases of malaria. Deaths related to malaria by clinicians in the health facilities were 685, out of which 290 deaths occurred among children under 5 years[3].

Transfusion transmitted malaria was discovered in 1884 when Gerhardt *et al* demonstrated on human subjects that the infection can be transmitted through blood innoculation[4]. A global review of world wide TTM recorded in 1911–1950 by Bruce-Chwatt reported 350 cases of accidentally induced human malaria out of which, 245 were well described malaria cases related to blood transfusion[5]. In a review of data from Africa, transmission of malaria has been reported to occur from infected whole blood or pack red blood cells, leucocytes, fresh plasma and platelets[6]. The symptoms of malaria may begin weeks or days after transfusion[7], which can present as life threatening disease[8] depending on the number of parasites in the inoculum especially for non-immune recipients[9].

The risk of TTM is associated with the inability to correctly diagnose infected donors especially those with the low parasitaemia, as well as the ability of the parasite to survive in stored blood units even after the storage process[10, 11].

Commonly used tests include microscopy and RDT. Microscopy is the gold standard for malaria diagnosis although its wide application is limited[12]. It is able to differentiate malaria species, quantify parasites and inexpensive to perform. But, microscopy requires well-trained, competent microscopist with effective quality control and quality assurance[13].

Malaria rapid diagnostic tests are cost effective, does not require skilled labour[14], but can give false negative and false positive results[15]. PCR is highly sensitive but has not been deployed extensively because of its cost and required expertise.

Apparent lack of knowledge, incorrect beliefs and wrong attitudes has contributed to the difficulty in malaria prevention and control. The influence of people’s knowledge, values, attitudes and practices are special to any particular community, and must be understood before incorporated into the design and implementation of malaria prevention and control programs[16].

In addition to investigating the prevalence of *Plasmodium* parasitaemia in blood donors, we investigated the knowledge, practices and attitudes of the hospital staffs pertaining to transfusion transmitted malaria.

## Methodology

### Study site

The study was conducted at the Seventh Day Adventist hospital, a public health care facility in Kumasi, Ghana. The SDA hospital has a total number of 84 patient beds with a Surgical unit, Maternity unit, Medical laboratory unit, Out-patient departments, Pharmacy department and a male and female wards. The hospital has a staff strength of 216 workers and a wide range of medical staff including consultants, senior medical doctors, specialists, junior medical doctors, interns and various categories of auxiliary nurses and nurse aids, pharmacy staff and Laboratory staff. About 400 blood transfusions are carried out each year in the hospital and the blood is mostly from family replacement and voluntary donors. The hospital has guidelines for routinely screening donor’s blood for Hepatitis B and C, Human Immune Deficiency Virus (HIV) and Syphilis. There are however no guidelines for malaria screening. After the collection of blood from the blood donors, blood grouping is performed and the blood is appropriately labeled and stored in fridges in the blood bank.

### Study design

This was a prospective cross sectional study and recruitment of subjects were done from September 2016 to April 2017. The study was conducted in two parts. The first part consisted of testing participant’s blood for *Plasmodium* parasites and the second part involved health workers answering a questionnaire. The study recruited a total of 200 subjects for *Plasmodium* testing (100 blood donors and 100 non-donors who served as controls) and 100 health workers to complete questionnaires.

Blood donors who came to the blood donation center at SDA hospital were approached for enrollment into the study. The non-blood donors (Healthy controls) were relatives who escorted patients into the hospital. They were approached at the Out-Patient Department. The inclusion criteria in this study for both donors and non-donors were persons aged between 15–60 years, a haemoglobin level of more than 12.5g/dl and being hepatitis B, hepatitis C, syphilis (VDRL) and HIV negative. Participants were excluded from the study if they fulfilled the inclusion criteria, but had signs of malaria or had been treated for malaria 3 weeks prior to enrolment. Written informed consent was obtained from eligible donors and non-donors. 3 weeks was an adequate time to allow clearance of antimalarials from the bloodstream of participants so as to rule out the possibility of encountering false negatives test results. Consent was also sought from parents or guardians of minors that qualified to participate in the study.

Health workers were approached individually to obtain their consent to participate in the study. They were contacted at the various wards in the hospital (males, females and children ward), consulting rooms, the dispensary rooms, pharmaceutical stores and the theatre. Participant’s information leaflets were given out to participants to briefly explain the project and also offered them the chance to ask questions.

### Test methods

1ml of venous blood sample was collected from both blood donors and non-donors. Blood samples were used for microscopy, RDT and blood grouping. For microscopy, 6ul of blood from blood donors and non-donors were used to prepare thick blood films and 2ul to prepare thin blood films. The smears were examined using the X100 objective lens after staining in a 1:10 Giemsa stain (ph 7.2).

Parasite count was determined by counting the number of parasites seen in relation to a predetermined number of white blood cells and an average of 8000/μl was taken as standard[17]. This is the usual way to quantitate *Plasmodium* parasites on a thick blood film[18].

$$\mathrm{NUMBER}\;\mathrm{OF}\;\mathrm{PARASITES}/\mu L=\frac{NUMBER\;OF\;PARASITES\;COUNTED}{NUMBER\;OF\;WHITE\;BLOOD\;CELLS\;COUNTED}\times 8000$$

5ul from each donor and non-donor blood samples were screened using the SD Bioline malaria Ag Pf test kit, a histidine rich protein (HRP-2) based rapid diagnostic test that detects *P*. *falciparum*, according to the manufacturer’s instructions[19].

Drops of the obtained blood samples from the participants were used to perform ABO phenotyping using the tile method[20].

### Questionnaire

One hundred (100) questionnaires were distributed to health workers who gave their consent to participate in the study. The questionnaire was an adaptation from one that had been used in a previous study[21]. It was categorized into three-parts with a socio-demographics section, knowledge section and a section on attitudes and practices.

### Ethical consideration

Ethical approval was obtained from the Committee on Human Research Publication and Ethics of the School of Medical Sciences, Kwame Nkrumah University of Science and Technology. Permission was also obtained from the hospital authorities prior to the conduct.

## Data analysis

Data obtained from this study was entered, coded in excel and exported into SPSS 10.17504/protocols.io.pxmdpk6 [PROTOCOL DOI]

## Results

### Demographics of blood donors and controls

Two hundred (200) participants were recruited for this aspect of the study, consisting of 100 blood donors and 100 non-donors. Majority of the participants were males (83%) in blood donors while females made up the majority (58%) in the non-donors. Participants aged 26–36 years made up the majority in both groups. Blood group O+ dominated in both groups and blood group B- being the least in blood donors (Table 1).

**Table 1:** Demographic characteristics of study participants.

	Characteristics	Blood Donors n = 100	Non-Donors n = 100
Gender	Male	83	42
	Female	17	58
AGE	15–25	32	23
	26–36	37	31
	37–47	28	22
	48–58	3	22
	59–60	0	2
BLOOD GROUP	A+	15	11
	B+	16	16
	B-	1	4
	AB+	7	4
	O+	59	57
	O-	2	8

### Malaria prevalence rate

Out of the 200 blood samples collected, the prevalence rate of *Plasmodium* parasitaemia in blood donors when tested by malaria RDTs and microscopy was 8% and 3% respectively with no statistical difference (X^2^ = -0.05, P = 0.101) between the methods used in detection of the *Plasmodium* parasites.

For non-donors, the prevalence rate determined by malaria RDT and microscopy was 5% and 2% respectively. There was also no statistical difference between the methods used in detection of the *plasmodium* parasites (X^2^ = -0.05, P = 0.743).

*Plasmodium falciparum* was the only species identified in both groups.

Majority [(37.5%) 3/8] of those with positive malaria RDT were in the age group of 15-25years and 26-36yrs in blood donors. In non-donors, majority of those with positive malaria RDT were aged 37-47years [40% (2/5)] (Table 2). Majority [66.7% (2/3)] of those with positive malaria microscopy were aged 37-47years in blood donors (Table 2). Males had a higher prevalence [100% (8/8)] by RDT than females in blood donors.

**Table 2:** Comparison of the prevalence rate of malaria using microscopy and RDT.

		Blood donor N = 100 (%)	Non donors N = 100 (%)
METHOD	RDT(Positives)	8(8.0)	5(5.0)
	Microscopy (MPS SEEN)	3(3.0)	2(2.0)
GENDER	RDT (Positives)	Number (%)	Number (%)
	Males	8 (100)	4 (80.0)
	Females	0(0.0)	1 (20.0)
	Microscopy (MPS SEEN)	Number (%)	Number (%)
	Males	2(67.7)	1 (50.0)
	Females	1 (33.3)	1 (50.0)
AGE GROUPS	RDT (Positives)	Number (%)	Number (%)
	15–25	3 (37.5)	1 (20.0)
	26–36	3 (37.5)	1 (20.0)
	37–47	2 (25.0)	2 (40.0)
	48–58	0(0)	1 (20.0)
	Microscopy (MPS SEEN)	Number (%)	Number (%)
	26–36	1 (33.3)	1(50.0)
	37–47	2 (66.7)	1 (50.0)

Blood group O+ recorded the highest prevalence in both groups, with a prevalence rate of 50% (4/8) and 100% (3/3) by malaria RDT and Microscopy respectively in blood donors. Blood groups O-, B- and A+ were all parasitaemia negative by both methods. There was no significant statistical difference between the detection of *Plasmodium* parasitaemia by the methods [RDT(X^2^ = -0.05, P = 0.470), Microscopy(X^2^ = -0.05, P = 0.828)] used in relation to the blood groups.

### Survey

100 questionnaires were distributed and collected for data entry and analysis.

#### Socio-demographics

Majority of the respondents were males (53%) and were aged between 20–30 years (76%). The age group 51–60 years and above 61 years had no respondents. Majority of the respondents were nurses (Table 3).

**Table 3:** Socio demographics of respondents (Health workers).

	Characteristic	Frequency	Percentage (%)
GENDER N = 100	Male	53	53.0
	Female	47	47.0
AGE N = 100	20–30	76	76.0
	31–40	18	18.0
	41–50	6	6.0
	51–60	0	0.0
	>61	0	0.0
JOB CATEGORY N = 100	Doctor	3	3.0
	Pharmacist	6	6.0
	Nurse	49	49.0
	Midwife	5	5.0
	Physician Assistant	6	6.0
	Biomedical Assistant	6	6.0
	Laboratory technician	7	7.0
	Others	18	18.0

#### Health workers knowledge about TTM

There were 74% (74/100) of respondents who had heard of TTM. Out of those 74 health workers who knew about TTM, 78.4% (58/74) thought that malaria could be transmitted by blood transfusion and 62.2% (46/74) regarded TTM as a life threatening disease (Table 4). Majority [64.9% (48/74)] indicated that, the number of malaria cases would reduce if malaria parasites in blood donors were eradicated. Concerning ways in which TTM could be reduced, 66.8% (32/48) wrongly assumed donor deferral and specific antimalarial antibody screening would be the best method in reducing TTM. Minority of the respondents (21%) also wrongly stated that the hospital’s blood bank screens blood donors for malaria parasites in addition to other transfusion transmitted infections. Out of 42 health workers, 40.5% (17/42) health workers indicated every individual stood at risk of acquiring TTM (Table 4).

**Table 4:** Knowledge of health workers about TTM.

Question	Options	Frequency	Percentage (%)
Heard of TTM	Yes	74	74.0
	No	26	26.0
Acquire malaria through Blood transfusion	Yes	58	78.4
	No	5	6.8
	Not sure	11	14.9
TTM is a serious and life threatening disease	Yes	46	62.2
	No	16	21.6
	Not sure	12	16.2
Most at risk group for TTM	Infant A	1	2.4
	Children B	12	28.6
	Pregnant women C	10	13.5
	Travelers D	0	0.0
	Patients with HIV/AIDS E	0	0.0
	Everyone F	17	40.5
	Non-immune migrant G	0	0.0
	BCD	1	2.4
	ABCDF	1	2.4
Does your blood bank screen donor blood for TTI including malaria	Yes	21	21.0
	No	33	33.0
	Not sure	46	46.0
Will eliminating malaria parasite in donors curtail the number of malaria cases?	Yes	48	64.9
	No	14	19.0
	Not sure	12	16.2
If yes, which of the following will you consider to reduce TTM?	Donor deferral and Specific antimalarial immunoglobulin screening	32	66.7
	Specific Donor Questioning	4	8.3
	Administration of antimalarials to blood units	10	20.8
	Specific donor questioning and administration of antimalarials to blood units	2	4.2

#### Attitudes and practices of health workers towards ttm

There were 74.3% (55/74) respondents who thought that screening for malaria parasites in blood donors was necessary in Ghana. Among the 100 respondents, 73%, 78% and 84% indicated their unwillingness to transfuse Syphilis, Hepatitis B, and Human Immuno Deficiency virus (HIV) positive blood respectively in case of emergency but 41% were willing to transfuse malaria in case of emergency. Among those willing to transfuse malaria positive blood, the major reason, [41.5% (17/41)] was because TTM was easy to treat. On the other hand, 62.2% (23/37) of the respondents were reluctant to transfuse malaria because of the risk of severe complications. A few respondents (5%) indicated that they had dealt with clinical issues of TTM. Rapid diagnostic test (44%) and microscopy (40%) were the most suitable screening method for TTM suggested by respondents. Among the reasons for the test method, 42.5% (17/40) indicated their willingness to use microscopy due to its high specificity and 47.7% (21/44) indicated their readiness to go for malaria RDT because it was easy to operate (Table 5).

**Table 5:** Attitude and practices of health workers towards TTM.

characteristics	Response	Frequency	Percentage
Is malaria screening prior blood donation necessary in Ghana?	Yes	55	74.3
	No	6	8.1
	Not sure	13	17.6
Will you transfuse Syphilis positive blood in case of emergency?	Yes	8	8.0
	No	73	73.0
	Not sure	19	19.0
Will you transfuse Hepatitis B positive blood in case of emergency?	Yes	4	4.0
	No	78	78.0
	Not sure	18	18.0
Will you transfuse HIV positive blood in case of emergency?	Yes	2	2.0
	No	84	84.0
	Not sure	14	14.0
Will you transfuse Malaria positive blood in case of emergency?	Yes	41	41.0
	No	37	37.0
	Not sure	22	22.0
If no for Syphilis, state why	There is no vaccine	9	12.3
	It has no cure	3	4.1
	High mortality	8	11.0
	Against medical practices	16	22.0
	Severe complication	28	38.3
	Don’t know for certain but I wont	9	12.3
If yes for Syphilis state why	It is safe to transfuse	1	12.5
	It is not fatal	6	75.0
	It is not part of the well-known TTI	0	0.0
	It is easy to treat	1	12.5
	It has a vaccine	0	0.0
	Don’t know for certain but I will	0	0.0
If no for Hepatitis B, state why	There is no vaccine	6	7.7
	It has no cure	9	11.5
	High mortality	19	24.4
	Against medical practices	15	19.2
	Severe complication	22	28.2
	Don’t know for certain but I wont	7	9.0
If yes for Hepatitis, state why	It is safe to transfuse	2	50.0
	It is not fatal	1	25.0
	It is not part of the well-known TTI	0	0.0
	It is easy to threat	0	0.0
	It has a vaccine	1	25.0
	Don’t know for certain but I will	0	0.0
	There is no vaccine	0	0.0
	It has no cure	0	0.0
	High mortality	2	5.4
	Against medical practices	6	16.2
	Severe complication	23	62.2
	Don’t know for certain but I wont	5	13.5
	Against medical practice and severe complication	1	3.0
If yes for malaria, state why	It is safe to transfuse	3	7.3
	It is not fatal	5	12.2
	It is not part of the well-known TTI	7	17.0
	It is easy to treat	17	41.5
	It has a vaccine	2	4.9
	Don’t know for certain but I will	4	9.8
	Safe to transfuse n easy to treat	3	7.3
Treated patients believed to be suffering for post-transfusion diseases with regards to TTM	Yes	5	5.0
	No	60	60.0
	Not sure	26	26.0
	Not applicable	9	9.0
If yes what were the symptoms	Chills	0	0.0
	Fever	2	40.0
	Sweating	0	0.0
	Headache	1	20.0
	Vomiting	0	0.0
	Others	0	0.0
	Fever, chills, headache and vomiting	1	20.0
	Fever and headache	1	20.0

## Discussion

In this study, which was conducted in the middle belt of Ghana, prevalence rate of malaria in blood donors by RDT was 8%. This prevalence is similar to an earlier study from Southern Ghana by Owusu-Ofori *et al* where the prevalence was 10% in both donors and non-donors[21]. Malaria prevalence rates in blood donors across Africa vary widely. The prevalence of malaria parasites in blood donors depends on the local endemicity and transmission season and varies from 0.6% in Kenya, Nairobi, which is not endemic for malaria, to over 50% in highly endemic northern Nigeria[22]. The World Health Organization recommends that all malaria endemic countries should screen donor’s blood for malaria parasites prior to blood transfusion[23]. Parasitaemia in asymptomatic donors is however usually low and will require a highly sensitive test to detect it. Asymptomatic parasitaemia may serve as a reservoir for continued transmission[24]. Malaria PCR has a higher sensitivity than microscopy and RDT but it is not widely available to be used as a screening tool in malaria endemic countries. The presence of parasitaemia in the blood donor represents a risk to the recipient of that blood. The risk of the recipient developing TTM is however dependent on other factors including the immune status as well as the parasite density. The prevalence rate of *Plasmodium* parasitaemia in males were more than half of females in blood donors. But in this study, it was found out, there was no significant difference between microscopy and RDT, in establishing the prevalence rate of *Plasmodium* parasitaemia in donors blood relating to gender (X^2^ = -0.05, P = 0.881). This is consistent with the findings of the study conducted by Ukaga *et al*[25]. who reported the prevalence rate of malaria to be 22.5% and 25.4% for males and females respectively in Nigeria, although the prevalence rate was not associated with gender. It is also consistent with Alli *et al*., study’s, who also reported that, the prevalence rate of malaria parasites in blood donors was not gender dependent (p = 0.535)[26].

From the present study in blood donors and as expected, blood group O+ was the majority group with a percentage frequency of 59.0%, and the least was blood group O- with a total of 2%. Study done by Maame Ama Wiredu *et al*., (2016) shows that, blood group O+ has the highest frequency of occurrence among the various ethnic groups in Ghana with the exception of the Guans[27]. The results obtained is also in agreement with reports from studies by Nag *et al*.,[28] and, Periyavan *et al*.,[29] The prevalence of *Plasmodium* infection was generally higher in blood group O+, (50%) by RDT and (100%) by microscopy followed by blood group AB+ and B+. The findings of this study was consistent with that of the findings of Tela *et al*[30]. but contrary to the findings of Pathirana *et al*[31]. which reported a low percentage of blood group O+ with *Plasmodium* parasitaemia and a higher percentage of blood group A with malaria parasites. Findings from the study conducted by Christian M and Walter Dzik indicated that the distribution of blood group O is higher in geographic regions, where malaria is previously or currently endemic like Ghana. This is because, blood group O offers a greater survival advantage than the other blood groups. So the chances of finding the ratio of blood group O to the other blood groups would be highest in regions where malaria is endemic[32].

Many of the health workers had heard about TTM but their knowledge can be approved as 66.8% of those who had heard about TTM wrongly assumed that donar-deferral and antibody screening would reduce TTM. Though malaria has been neglected as one of the TTI, majority of the health workers had heard about it. However a few of the respondents wrongly assumed that, donor blood screening for malaria parasites was performed in the hospital. The health workers probably presumed that because routine screening takes place for HIV, Hepatitis B and C, malaria screening would be included.

Many health workers were reluctant to transfuse HIV/AIDS, Hepatitis B and Syphilis positive blood in case of emergency. Majority of the respondents indicated that this was due to severe complications associated with the diseases. They were however willing to transfuse malaria positive blood if it was an emergency. Out of 41 respondents, 41.5% (17/41) were willing to transfuse malaria positive blood because malaria was easy to treat. In a previous study, no reasons was given for their willingness to transfuse malaria positive blood.

Most respondents recommended RDT (HRP-2) for screening for *Plasmodium* parasites in blood donors, because it was easy to operate. RDT is considered as one of the point of care tests[33] and thus is accessible to most health workers. Most health workers would prefer to use it instead of microscopy.

Health workers who selected microscopy as the screening test for malaria parasites indicated that, they recommended it due to its high specificity. These indicate that, the health workers have a good knowledge regarding malaria test methods.

Interestingly only 5% of the respondents suggested PCR as a screening test for *Plasmodium* parasites in blood donors. PCR for *Plasmodium* surpasses both microscopy and RDT in both sensitivity and specificity[22]. It may be that, the reason PCR was selected by only a few workers was because it is expensive and requires highly skilled personnel to operate it.

Half of the respondents (64%) knew malaria cases would reduce, if malaria parasites in blood donors were eliminated. This source of knowledge could be due to published articles and malaria campaigns going on at Kwadaso. It was interesting to find that only 5% of respondents had treated TTM. This is very low indeed and may be an indication of the fact that not many healthworkers look out for it.

Out of 48 respondents, 66.8% (32/48) indicated donor deferral and specific antimalarial antibody screening to reduce TTM. Donor deferrals due to visits to malaria endemic areas in combination with screening for specific antimalarial immunoglobulin provides an effective means of reducing the risk of TTM in non-endemic countries[11]. This is however not applicable in malaria endemic regions. Asymptomatic malaria cannot be identified by history or examination and therefore blood screening methods have to be explored if parasitaemia is to be determined.

The study showed that majority of the blood donors were males (83%). In this study, the ratio of male to female blood donors (83:17) was similar to the study conducted by Tagny *et al*[34] who reported 61% of donor population to be males in Togo and 71.2% in blood donors in Burkina Faso. Owusu-Ofori and Gadzo[21] also reported 55% of donors to be males.

## Conclusion

The prevalence rate of *Plasmodium* parasitaemia in blood donors at SDA hospital was low. The prevalence in blood donors by RDT and microscopy was 8% and 3% respectively and by RDT and microscopy in controls was 5% and 2% respectively.

The knowledge of the health workers was good. Most health workers (78.4%) knew malaria could be acquired through blood transfusion. 74.3% (55/74) of the health workers were aware that malaria screening prior blood donation was necessary. Majority of the health workers were willing to transfuse malaria positive blood in case of emergency and 41.5% indicated their willingness to do so because it was easy to treat.

## Recommendation

More in-service training and seminars are recommended for health workers to improve their knowledge with regards to TTM as to equip them with the best attitude and practices towards it.

Blood transfusion services and malaria control prgrammes should collaborate with hospitals to come up with evidence-based policies that will guide how *Plasmodium falciparum* blood donors and the donated blood should be managed.

## Supporting information

S1 File (PDF)

S2 File (XLSX)
