# Supplementary material for: Prevalence of Plasmodium parasitaemia in blood donors and a survey of the knowledge, attitude and practices of transfusion malaria among health workers in a hospital in Kumasi, Ghana
Source: PLoS One. 2018 Nov 5;13(11):e0206303. doi: 10.1371/journal.pone.0206303 (PMC6218034; doi:10.1371/journal.pone.0206303)
Supplement: S1 File — (PDF) [file pone.0206303.s001.pdf]

QUESTIONNAIRES FOR MEDICAL STAFFS ON TRANSFUSION TRANSMITTED MALARIA

Section A

(SOCIO-DEMOGRAPHICS).

i) **Gender:** Male [ ] Female [ ]

ii) **Age**: 20-30 [ ] 31-40 [ ] 41-50 [ ] 51- 60 [ ] 61 and above [ ]

iii). **Job Category**

Medical Director [ ] Doctor [ ] Pharmacist [ ] Biomedical Scientist [ ] v) Laboratory Technician [ ] Nurse [ ] Midwifes [ ]

Physician Assistant [ ] Other (specify)………………………

1 (**HEALTH WORKERS KNOWLEDGE ABOUT TRANSFUSION TRANSMITTED MALARIA (TTM)).**

i) **Have you heard of TTM?** YES[ ] NO[ ]

If YES, please proceed to answer question 1(ii) to 1(vii), if NO, please skip to questions under (2)

ii) **Can one acquire malaria through blood transfusion?** YES [ ] NO[ ] NOT SURE[ ]

iii) **Is TTM a serious and life threatening disease?** YES[ ] NO[ ] NOT SURE[ ]

iv) **Does every patient/recipient stand a risk of acquiring malaria through blood transfusion?** YES[ ] NO[ ] NOT SURE[ ]

v) If yes, who are the most at risk group for TTM?

Infants [ ] Children below 5 years[ ] Pregnant women[ ] Travelers[ ]

Patients with HIV/AIDS[ ] Everyone[ ] None immune- migrants[ ]

Other(Specify)……………………………...

If no, can you state why?

…………………………………………………………………………………………

……………………………………………………………………………………....

vi) **Does your blood bank screen donor blood for Transfusion Transmitted infections including malaria** YES [ ] NO [ ] NOT SURE[ ]

vii) **Will eliminating malaria parasites in blood donors help curtail the number of malaria cases in Ghana?** YES [ ] NO[ ] NOT SURE[ ]

If YES, which of the following ways will consider the best for reducing TTM ?

Donor deferral and Specific antimalarial immunoglobulin screening [ ]

Specific donor questioning [ ]

Administration of anti-malaria drugs to transfusion [ ]

Other(specify)…………................... ………………………………….

2 **(ATTIUDES AND PRACTISES TOWARD TRANSFUSION TRANSMITTED MALARIA OF HEALTH WORKERS.)**

i) **Is malaria screening prior to blood donation necessary in Ghana?** YES[ ] NO[ ] NOT SURE[ ] SURE[ ]

ii) **Will you transfuse the following positive transmissible infections in case ofemergency?**

SYPHILS YES[ ] NO[ ] NOT SURE[ ] HEPATITIS B YES[ ] NO[ ] NOT SURE[ ]

HIV YES[ ] NO[ ] NOT SURE[ ] MALARIA YES[ ] NO[ ] NOT SURE[ ]

iii)**If NO for syphilis, state why**

There is no vaccine[ ] It has no cure[ ] High mortality[ ] Against Medical Practice [ ]

Severe complications[ ] Don’t know for certain but I won’t[ ]

Other (specify) ………………………………………………………………………………

If YES for syphilis state why

It’s safe to transfuse [ ] It’s not fatal [ ] Its not part of the well known transfusion transmitted Infections [ ] It’s easy to treat [ ] It has a vaccine [ ] Don’t know for certain but I will[ ] Other(specify)[ ]……………………………………………………………………..

vi)**If NO for Hepatitis B state why**

There is no vaccine[ ] It has no cure[ ] High mortality[ ] Against Medical Practice [ ]

Severe complications[ ] Don’t know for certain but I won’t[ ]

Other (specify) ………………………………………………………………………………

If YES for Hepatitis B state why

It’s safe to transfuse [ ] It’s not fatal [ ] Its not part of the well known transfusion transmitted Infections [ ] It’s easy to treat [ ] It has a vaccine [ ] Don’t know for certain but I will[ ] Other(specify)[ ]……………………………………………………………………..

v) **If NO for malaria state why**

There is no vaccine[ ] It has no cure[ ] High mortality[ ] Against Medical Practice [ ]

Severe complications[ ] Don't know for certain but I won't[ ]

Other (specify) ……………………………………………………………………………

If YES for malaria state why

It's safe to transfuse [ ] It's not fatal [ ] Its not part of the well known transfusion transmitted Infections [ ] It's easy to treat [ ] It has a vaccine [ ] Don't know for certain but I will[ ] Other(specify)[ ]……………………………………………………………………..

vi) **Have you treated a patient believed to be suffering from post-transfusion diseases with regards to TTM?** YES [ ] NO [ ] NOT SURE[ ] NOT APPLICABLE[ ]

If Yes, what were the symptoms?

Chills[ ] Fever[ ] Sweating[ ] Headache[ ] Vomiting[ ] Others (specify)……………………………………………………………………………........................

v) Which of the following methods would you consider advisable for screening malaria parasites in blood donor's prior transfusion?

Microscopy[ ] Polymerase Chain Reaction[ ] Rapid Diagnostic Test[ ]

Enzyme Immunoassay[ ] Other (specify)……………………………………………

vi)**Which of the following properties will you consider in choosing your answer in (v)**

High sensitivity[ ] low sensitivity[ ] High specificity[ ] low specificity[ ] Easy to operate[ ] Economic[ ]
